# Parafibromin as a Diagnostic Instrument for Parathyroid Carcinoma-Lone Ranger or Part of the Posse?

**DOI:** 10.1155/2010/324964

**Published:** 2010-12-12

**Authors:** C. Christofer Juhlin, Anders Höög

**Affiliations:** ^1^Department of Molecular Medicine and Surgery, Karolinska Institute, Karolinska University Hospital Solna, 17176 Stockholm, Sweden; ^2^Department of Oncology-Pathology, Karolinska Institute, Karolinska University Hospital Solna, 17176 Stockholm, Sweden

## Abstract

The diagnosis of parathyroid carcinoma requires an invasive growth pattern or metastases detected at histopathological examination; unfortunately, not all carcinomas exhibit visible malignant properties at the initial assessment. Therefore, immunohistochemical markers have been sought for the recognition of parathyroid malignancy. In 2003, the *Hyperparathyroidism 2 (HRPT2)* gene was found mutated in the majority of sporadic parathyroid carcinomas investigated, and studies regarding the protein product parafibromin proposed loss of nuclear parafibromin as a highly sensitive marker for the detection of parathyroid carcinoma. Recent studies have not fully reproduced these findings, as subsets of carcinomas display positive parafibromin immunoreactivity, and fractions of adenomas demonstrate absent expression. Overall, parafibromin is a marker of value to the endocrine pathologist, but it cannot be recommended as a sole indicator of parathyroid carcinoma. Additional markers such as protein gene product 9.5 (PGP9.5) and adenomatous polyposis coli (APC) could complement parafibromin when assessing malignant potential of parathyroid tumours.

## 1. Introduction

Primary hyperparathyroidism (PHPT) originating from a parathyroid carcinoma is an infrequent finding in the clinical setting. Clinical manifestations indicative of parathyroid carcinoma include profound hypercalcemia with severe metabolic disease and a palpable mass in the anterior neck region [1]. Histopathological features such as nuclear atypia, macronucleoli, fibrous bands splitting the parenchyma, trabecular growth, and elevated mitotic counts are common findings in parathyroid carcinomas, but are also found in various proportions of parathyroid benign tumours [2]. Although the above-mentioned observations might suggest parathyroid carcinoma, the definite diagnosis is based on the histopathological identification of malignant properties such as vascular or perineural invasion, alternatively direct spread into surrounding local tissues, as well as the observation of distant metastases [3]. The diagnosis of parathyroid carcinoma cannot be established at preoperative investigation, since fine needle cytology should be avoided if a malignant parathyroid lesion is suspected. Furthermore, the diagnosis cannot easily be established interoperatively by frozen section analysis due to the suboptimal morphology obtained. Instead, the definite diagnosis is either made at routine histopathological examination after the initial parathyroidectomy, or years after the initial surgery when the patient presents with clinical manifestations of metastases. If diagnosed directly postoperatively, the treating physicians are often obliged to choose between an aggressive reoperation and vigilant followup.

Optimally, the diagnosis of a malignant tumour is made prior to the onset of advanced malignant properties so that the tumour can be radically removed before an eventual metastatic spread. Extensive research has, therefore, been conducted to identify a potential molecular marker which could discriminate between malignant and benign parathyroid tumours prior to the onset of required histopathological requirements. Early studies focused on immunohistochemical analyses of well-established proteins controlling the cell cycle process as well as apoptosis, such as the retinoblastoma protein (pRb), p53, and cyclin D1 [4–6]. Although initially promising, subsequent studies have reported substantial overlap between malignant and benign tumours, thereby reducing the overall specificity of the method for each of these proteins [7–9]. The proliferation marker Ki-67 has also been implicated as an adjunct tool, since parathyroid carcinomas generally have greater Ki-67 counts than adenomas [10]. Although an overlap between the two groups exists, the current WHO classification guidelines concerning parathyroid carcinoma suggests that tumours with Ki-67 counts greater than 5% should be subject to closer followup due to an increased risk of malignancy [3, 7]. 

**Figure 1:**
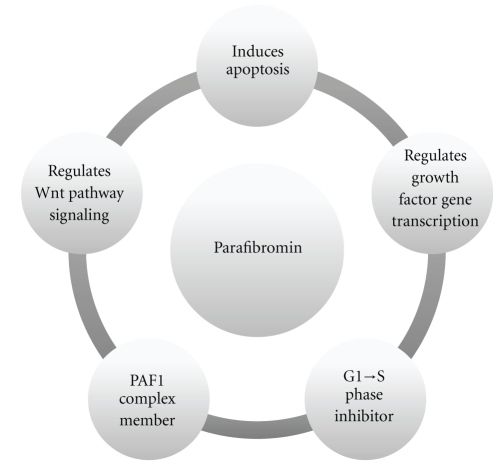
Schematic overview regarding parafibromin functions. Parafibromin is a tumour-suppressor protein which regulates apoptosis, cell-cycle transition, growth factor gene expression (such as insulin-like growth factors I and II), the tumour-associated wingless type (Wnt) pathway, as well as the polymerase associated factor 1 (PAF1) complex. Loss of parafibromin expression through mutational inactivation of the *HRPT2* gene could in theory affect one or several of these molecular branches, which in turn would propagate parathyroid tumorigenesis.

In 2002, Carpten et al. demonstrated that the hyperparathyroidism-jaw tumour (HPT-JT) syndrome is caused by a germline mutation in the tumour suppressor gene *Hyperparathyroidism 2* gene (*HRPT2*), located at 1q25-q31 [11]. Interestingly, a subset of HPT-JT kindred develops malignant parathyroid tumours, and subsequent analyses of sporadic parathyroid tumours identified somatic *HRPT2* mutations in the majority of malignant cases examined [12, 13]. This is opposed to the findings in adenomas, where *HRPT2* mutations only have been detected in small subsets of cases [11]. *HRPT2* encodes a ~60 kDa protein termed parafibromin, a protein which bears sequence resemblance with the yeast protein Cdc73 [11]. Just like its yeast counterpart, parafibromin has been shown to be a member of the polymerase associated factor 1 (PAF1) complex involved in histone ubiquitination and methylation, resulting in chromatin remodeling which in turn acts as a regulatory mechanism of gene transcription [14, 15]. In addition, parafibromin has been endowed with tumour-suppressive functions such as inducing apoptosis, inhibiting G1 to S phase transition of the cell cycle, regulating the wingless type (Wnt) canonical pathway, as well as directly regulating growth factor gene expression by binding to their gene promoters [16–19] (Figure 1). It is, therefore, expected that loss of nuclear parafibromin through mutational inactivation of *HRPT2* will augment cellular proliferation. Following the initial discovery of inactivating *HRPT2* mutations in parathyroid carcinomas, studies were launched with the aim of characterizing parafibromin expression in parathyroid tumours. In the first study published by Tan et al. in 2004, the authors constructed a monoclonal parafibromin antibody (2H1) and investigated a large number of tumours for parafibromin expression using immunohistochemistry [20]. The authors observed complete absence or focal loss (mixed pattern of positive and negative nuclei) of parafibromin nuclear immunoreactivity in the vast majority of parathyroid carcinomas. The findings resulted in an overall 96% sensitivity and 99% specificity in diagnosing parathyroid carcinoma using parafibromin, as the 48 sporadic adenomas investigated exhibited retained expression. The study also implemented 9 adenomas from HPT-JT kindred with established *HRPT2* mutations; 6 (67%) of these cases displayed total loss and 2 cases exhibited focal loss (22%) whereas one case was positive for parafibromin expression (11%). These findings suggest that the absence of parafibromin immunoreactivity might be indicative of *HRPT2* mutations, which is further supported by the loss of parafibromin expression in the majority of carcinomas, a group known to frequently carry *HRPT2* inactivating mutations.

The findings of Tan et al. were confirmed by an independent study from Australia, in which the authors employed the 2H1 antibody and observed loss of parafibromin immunoreactivity in the majority of sporadic carcinomas (8/11; 73%) and HPT-JT-related tumours (3/4; 75%) as opposed to positive staining in benign tumours [21]. The sensitivity was lower (76%) as opposed to 96% in the previous study of Tan et al., and one of the reasons for this divergence was due to the fact that Gill et al. only classified cases with complete absence of nuclear immunoreactivity as negative whereas cases with “weak staining” (similar to the term “focal loss” proposed by Tan et al.) were categorized as positive. Although reducing the overall sensitivity for the detection of parathyroid carcinomas, the authors state that this system was selected to lessen the rate of interobserver error when interpreting a case with focal loss. As a result, the authors advocate that complete absence of parafibromin nuclear immunoreactivity is diagnostic for a parathyroid carcinoma or an HPT-JT-related tumour in the presence of positive internal controls. The authors conclude that the phenomenon of focal loss warrants further studies before it can be implemented in the diagnostic arsenal [21].

In a subsequent study, the authors assessed parafibromin immunoreactivity in a set of parathyroid tumours using monoclonal and polyclonal parafibromin antibodies targeting diverse epitopes scattered across the protein, including the previously used monoclonal parafibromin antibody 2H1 [22]. Reduced parafibromin expression was observed in the majority of parathyroid carcinomas whereas all adenomas displayed positive nuclear immunoreactivity. The results were comparable using all four parafibromin antibodies, and peptide neutralization assays confirmed the specificity of the obtained signals. Out of the 15 carcinoma cases with reduced or absent parafibromin immunoreactivity, only one sample displayed complete absence of expression whereas the remaining 14 cases exhibited partial loss (similar to the term “focal loss” proposed by Tan et al.) Interestingly, 3 out of 6 cases with established *HRPT2* mutations displayed positive parafibromin expression, suggesting that cases with *HRPT2* aberrations, not as a rule, display alterations in parafibromin immunoreactivity [22].

The discussion regarding the value of parafibromin as a discriminating marker took a new turn when a subsequent study showed excellent correlations between loss of parafibromin expression and *HRPT2* gene mutations in which 21/22 sporadic adenomas were parafibromin positive whereas all 11 carcinomas were negative for parafibromin immunoreactivity using the 2H1 antibody [23]. The parafibromin-negative adenoma and all carcinomas displayed truncating *HRPT2* gene mutations. To counter these findings, another group has since detected positive parafibromin expression in the majority of parathyroid carcinomas stemming from secondary hyperparathyroidism [24], and an additional study only observed complete loss of parafibromin expression in a third of parathyroid carcinoma specimens examined [25].

## 2. Discussion

*HRPT2* gene aberrancies play an important role in the progression of parathyroid malignancy, and *HRPT2* inactivating mutations have been established as a major event in sporadic parathyroid carcinomas [12, 13]. Subsequent studies regarding parafibromin expression in parathyroid tumours have reached consensus in that sense that a majority of parathyroid carcinomas display reduced or absent immunoreactivity as compared to normal parathyroid tissue or parathyroid adenomas [20–23, 25]. Therefore, the sensitivity for the method is validated and should be considered as quite high. The specificity seems to be exceptionally high, based on the findings of positive nuclear expression in the vast majority of parathyroid adenomas investigated. However, the overall specificity unfortunately does not seem to reach 100%, as a small fraction of seemingly sporadic, parathyroid adenomas without signs of atypia or relapsing disease apparently harbor *HRPT2* gene mutations and absent parafibromin expression using Western blot analysis as well as immunohistochemistry [26]. Although these adenomas were shown to exhibit cystic features, a feature commonly observed in the HPT-JT syndrome, the lack of germline *HRPT2* gene alterations in these cases suggest that they have evolved sporadically. In addition, independent studies have also demonstrated reduced parafibromin immunoreactivity and/or *HRPT2* mutations in a few cases of sporadic, parathyroid adenomas [23, 27]. These findings imply that *HRPT2* mutations and downregulation of parafibromin are present in very small subgroups of parathyroid benign tumours.

Since parathyroid carcinomas are so uncommonly observed in the clinical settings as opposed to parathyroid adenomas, parafibromin immunohistochemistry will require almost near-perfect specificity to reduce the number of false positive cases. With the currently reported specificity, even a few parathyroid adenomas with negative parafibromin staining will outrank the “true” parafibromin negative cases in the infrequently encountered carcinoma group. As a consequence, loss of parafibromin immunoreactivity could either imply parathyroid adenoma or carcinoma, but may also indicate an underlying *HRPT2* gene mutation [22]. Positive expression, however, strongly suggests a benign tumour. Given the fact that subsets of patients with seemingly sporadic parathyroid carcinoma have been found to carry germline inactivating mutations of the *HRPT2* gene [13], an observation of reduced parafibromin expression in a tumour sample could motivate *HRPT2* mutation analysis in the afflicted patient to exclude a possible hereditary background. This demonstrates one of the main benefits of utilizing parafibromin immunohistochemistry as a marker in the clinical context.

Another issue regarding parafibromin immunohistochemistry stems from the different staining patterns detected when analyzing parathyroid malignant tumours. Variations in the number of tumour cells with parafibromin expression vary between studies, with authors reporting total absence as well as a mixed pattern of positive and negative nuclei in addition to 100% positive nuclei in different proportions. For example, Gill et al. classified parathyroid tumours with focal loss as positive and, therefore, only approved a total absence of parafibromin immunoreactivity as diagnostic of malignancy [21]. However, in independent studies, approximately half of the parathyroid carcinomas analyzed displayed reduced expression rather than total absence of parafibromin, and the authors suggest that reduced expression of parafibromin as well as total absence of immunoreactivity indicates malignancy [20, 22]. The observed discrepancies between the staining patterns could possibly be explained by differences in the sample selection process, as some groups analyze carcinomas based on the WHO histopathological criteria and others based on biological evidence of malignant behavior (recurrences, metastases) [20–22]. Differences in the immunohistochemical methodology should also be considered, since parafibromin immunohistochemistry is influenced by a number of factors, including antigen retrieval time, antibody dilution, antibody incubation time, and type of antibody [22]. Another factor which might complicate the interpretation of parafibromin immunohistochemistry is the notion that a fraction of the established *HRPT2* germline and somatic mutations is of missense type rather than the more commonly observed nonsense or frameshift types [28]. Missense mutations would theoretically produce full-length parafibromin although functionally defective. Therefore, it would seem possible to obtain positive parafibromin immunoreactivity in subsets of *HRPT2* mutated cases if the antibody is not targeting the epitope of the corresponding missense alteration.

As a result of the reduced sensitivity and specificity of parafibromin as well as the different staining patterns observed, additional molecular markers have been assessed to complement parafibromin in the screening process. For example, protein gene product 9.5 (PGP9.5), encoded by the *ubiquitin carboxyl-terminal esterase L1* (*UCHL1*) gene has been shown to be upregulated in the majority of parathyroid carcinomas based on gene expression profiling, and the results were verified by immunohistochemistry [29]. When analyzing a large number of parathyroid carcinomas, positive staining for PGP9.5 was shown to bear a greater sensitivity for the detection of malignant behavior compared to that of parafibromin, while maintaining the high specificity. Another study suggested the implementation of the Wnt pathway tumour-suppressor adenomatous polyposis coli (APC) as an additional marker for the detection of parathyroid carcinoma, as negative immunoreactivity was demonstrated in 9 out of 12 parathyroid carcinomas whereas the expression was retained in all adenomas investigated [30]. Subsequent APC analyses on *HRPT2* mutated parathyroid adenomas with loss of parafibromin expression have shown that APC is uniformly expressed in these tumours, thereby demonstrating a superior specificity which potentially could be of clinical use [31]. In addition, galectin-3 and human telomerase reverse transcriptase (hTERT) constitute two other markers of promising value which could be of clinical importance when assessing parathyroid tumours of uncertain malignant potential [32, 33].

In present time, several pathology departments are known to habitually perform parafibromin immunohistochemistry for parathyroid tumours which are not clearly benign or that display atypical findings at the histopathological examination. Positive parafibromin expression clearly points towards benign disease, and reduced parafibromin immunoreactivity should probably indicate intensified followup, since this pattern is often encountered in parathyroid malignant disease. In addition, reduced parafibromin expression could indicate *HRPT2* gene aberrancies that could motivate germline *HRPT2* testing to exclude possible familial disease. However, no clear consensus exists on how the different staining results should be interpreted (reduced expression versus total loss) as well as how this interpretation should influence the overall treatment of the afflicted patient. Given the current literature, routine histopathology combined with immunohistochemical analysis of parafibromin cannot alone be recommended as a definite screening method for parathyroid malignancy, but should probably be regarded as an aiding tool for the pathologist when assessing parathyroid tumours which are not clearly benign. Additional discovered or yet undiscovered markers are likely to increase the sensitivity and specificity for the proper recognition of parathyroid carcinomas in the clinical setting.
